# Novel Metabolic Approaches Targeting Cancer Cells

**DOI:** 10.3390/cancers15225448

**Published:** 2023-11-16

**Authors:** Anna Sebestyén, Michael P. Lisanti, Bela Ozsvari

**Affiliations:** 1 Department of Pathology and Experimental Cancer Research, Semmelweis University, 1085 Budapest, Hungary; sebestyen.anna@med.semmelweis-univ.hu; 2 Translational Medicine, School of Science, Engineering and the Environment (SEE), University of Salford, Manchester M5 4WT, UK; michaelp.lisanti@gmail.com

Metabolic reprogramming is one of the main hallmarks of cancer. Numerous studies have shown that the metabolic phenotype of cells within tumors is heterogeneous and distinct from their normal counterparts [1]. Furthermore, an altered metabolism has been recognized as one of the major mechanisms of resistance to current therapies. Therefore, targeting the metabolic differences between tumor and normal cells holds promise as a novel anticancer strategy.

This Special Issue, “Novel Metabolic Approaches Targeting Cancer Cells”, aims to compile the latest research on tumor cell metabolism and its impact on the development of novel therapeutic targets. The Special Issue contains five articles, two of which are reviews and three of which are original research papers from diverse research groups with remarkable contributions to the metabolism field in cancer.

Extracellular vesicles (EVs) are membranous vesicles delimited by a lipid bilayer without replicative capacity. They can range in size from 30 to 1000 nm, are generated by all cells, and have been found in multiple biofluids [2]. They are recognized as key players in intercellular communications and mediate many of the hallmarks of cancer via the delivery of diverse biological cargo molecules.

Carles-Fontana et al. explore the different EV-mediated mitochondrial reprogramming mechanisms supporting cancer survival and progression in their review. Firstly, they detail the main steps in EV biogenesis, release, and cargo in cancer development. Then they summarized a number of EV studies in which the EV-mediated modulation of mitochondrial processes occurred in cancer. Publications about EV cargos carrying various miRNAs derived from cancer cells which were shown to trigger metabolic coupling in the neighboring fibroblasts, or vice versa, are listed. Other EVs can initiate chemotherapy resistance, escape from immune response, or the activation of invasion and the metastasis of tumor cells through the modulation of OXPHOS, the reduction of Bax, and/or an increase in Bcl-2 expression in mitochondria. Cancer-associated fibroblasts have also been shown to transfer mtDNA to breast cancer cells via EV cargos [3]. At the end of their review, the authors list the uses of various modified EVs as delivery vectors for therapeutic cargoes, targeting mitochondria with miRNAs, and in different chemotherapeutic drugs like doxorubicin, paclitaxel, or even with enterotoxins.

Mátyási et al. investigate how vesicular NME1 and NME2 released by breast cancer cells influence the tumor microenvironment and its metabolism. After overexpressing these two metastasis suppressor genes in human invasive breast carcinoma cells, they found that NME1 was present in medium-sized EVs or microvesicles, whereas NME2 was abundant in both microvesicles and small-sized EVs or exosomes, as well. When human skin-derived fibroblasts were treated with NME1 or NME2 containing EVs, the expression of fatty acid and cholesterol metabolism-related genes decreased significantly in response to NME1 or NME2 containing EV treatment, which showed that these EV-mediated metastasis suppressors directly modify lipid metabolism in fibroblasts in the tumor microenvironment.

Oncometabolites are metabolites that accumulate in tumors as a consequence of an alteration in cellular respiration in metabolically rewired cancer cells. This process is considered a hallmark of cancer, driving cancer progression and therapeutic resistance [4].

Low-glycolytic tumors seem to utilize carbon sources other than glucose for bioenergetics and tumor proliferation [5]. Citrate is a well-known tricarboxylic acid (TCA) cycle intermediate and an important carbon source for the biosynthesis of fatty acids and cholesterol synthesis [6]. Kim et al., in their manuscript, examined the expression of various solute carrier protein (SLC) transporters in human hepatocellular carcinomas and investigated whether extracellular nutrient administration related to SLCs in low-glycolytic tumors can prevent hypoxic cancer progression. Their results showed that an increased citrate uptake was associated with decreased glucose uptake in in vitro cancer cell lines, mouse xenograft models, and human hepatocellular carcinomas. More importantly, they found a significant negative correlation between the sodium/citrate cotransporter and HIF1α expression, suggesting that citrate can be a potential therapeutic strategy preventing metabolic adaptation to hypoxic tumors. They showed that extracellular citrate treatment induced the failure of metabolic adaptation to hypoxia and tumor growth inhibition, which can be a potential therapeutic strategy in hepatocellular carcinomas.

Another important oncometabolite in the TCA cycle which has gained attention recently is succinate [7]. Casas-Benito et al. summarize the role of succinate in tumor development and detail the novel approaches targeting aberrant succinate metabolism. In the first part of the manuscript, they detail the history, main causes, and main characteristics of the Warburg effect and list the classical approaches targeting the Warburg Effect in cancer. They presented that the various effects of succinate accumulation related to cancer, which is mainly exerted through binding to its membrane receptor, G-protein coupled receptor 91 (GPR91)/succinate receptor 1 (SUCNR1), or by participating in mitochondrial ROS production. At the end of their review, they describe why succinate could be a novel target in cancer metabolism, highlighting the inhibition of succinate receptor 1 (SUCNR1), succinyl-CoA synthetase, TRAP-1, or the stimulation of SIRT3 expression as possible therapeutic targets.

Cancer-associated cachexia, defined as involuntary weight loss and breakdown of adipose and muscle tissue, is present in most patients with pancreatic cancer and contributes significantly to mortality [8]. Coleman et al. investigated whether β-hydroxy-β-methylbutyrate, a metabolite of leucine, could augment therapy in pancreatic cancer patients. To test this hypothesis, they used a diet-induced obese mouse model, subcutaneously injected with Panc02 tumor cells, with half of the received β-hydroxy-β-methylbutyrate and/or gemcitabine intraperitoneal treatment, as well. Tumor growth, muscle mass, and tumor immune profiles were monitored during the treatments. HMB supplementation promoted a significantly increased muscle fiber size, and suppressed inflammatory signaling, which also synergized with anti-PD1 immunotherapy and promoted the M1-like polarization of macrophages. Based on their findings, they showed that the use of β-hydroxy-β-methylbutyrate as a dietary supplement can promote immunosurveillance in PDAC tumors and protect muscle.

In summary, manuscripts published in this Special Issue describe how extracellular vesicles transmit essential factors among cells directly affecting the metabolism of the tumor and its microenvironment, and they can also serve as cargos to deliver various small molecules to target cancer cells. Other papers in this collection highlight the importance of oncometabolites, where the inhibition of their aberrant production can be the potential target of novel metabolic therapies, or where the accumulation of certain metabolites can help to induce the failure of metabolic adaptation in the tumor.

## References

[1] Martinez-Outschoorn U.E., Peiris-Pages M., Pestell R.G., Sotgia F., Lisanti M.P. (2017). Cancer metabolism: A therapeutic perspective. Nat. Rev. Clin. Oncol..

[2] Bebelman M.P., Smit M.J., Pegtel D.M., Baglio S.R. (2018). Biogenesis and function of extracellular vesicles in cancer. Pharmacol. Ther..

[3] Sansone P., Savini C., Kurelac I., Chang Q., Amato L.B., Strillacci A., Stepanova A., Iommarini L., Mastroleo C., Daly L. (2017). Packaging and transfer of mitochondrial DNA via exosomes regulate escape from dormancy in hormonal therapy-resistant breast cancer. Proc. Natl. Acad. Sci. USA.

[4] DeBerardinis R.J., Lum J.J., Hatzivassiliou G., Thompson C.B. (2008). The Biology of Cancer: Metabolic Reprogramming Fuels Cell Growth and Proliferation. Cell Metab..

[5] Liberti M.V., Locasale J.W. (2016). The Warburg Effect: How Does it Benefit Cancer Cells?. Trends Biochem. Sci..

[6] Martínez-Reyes I., Chandel N.S. (2020). Mitochondrial TCA cycle metabolites control physiology and disease. Nat. Commun..

[7] Kuo C.-C., Wu J.-Y., Wu K.K. (2022). Cancer-derived extracellular succinate: A driver of cancer metastasis. J. Biomed. Sci..

[8] Mueller T.C., Burmeister M.A., Bachmann J., Martignoni M.E. (2014). Cachexia and pancreatic cancer: Are there treatment options?. World J. Gastroenterol..

